# Importance of GFAP isoform‐specific analyses in astrocytoma

**DOI:** 10.1002/glia.23594

**Published:** 2019-01-22

**Authors:** Emma J. van Bodegraven, Jessy V. van Asperen, Pierre A.J. Robe, Elly M. Hol

**Affiliations:** ^1^ Department of Translational Neuroscience, Brain Center Rudolf Magnus University Medical Center Utrecht, Utrecht University Heidelberglaan 100, 3584 CX Utrecht The Netherlands; ^2^ Department of Neurology and Neurosurgery, Brain Center Rudolf Magnus University Medical Center Utrecht, Utrecht University Heidelberglaan 100, 3584 CX, Utrecht The Netherlands; ^3^ Netherlands Institute for Neuroscience, An Institute of the Royal Netherlands Academy of Arts and Sciences Meibergdreef 47, 1105, BA, Amsterdam The Netherlands

**Keywords:** astrocytoma, biomarker, GFAP, GFAP variants, GFAPδ, glioma, intermediate filaments

## Abstract

Gliomas are a heterogenous group of malignant primary brain tumors that arise from glia cells or their progenitors and rely on accurate diagnosis for prognosis and treatment strategies. Although recent developments in the molecular biology of glioma have improved diagnosis, classical histological methods and biomarkers are still being used. The glial fibrillary acidic protein (GFAP) is a classical marker of astrocytoma, both in clinical and experimental settings. GFAP is used to determine glial differentiation, which is associated with a less malignant tumor. However, since GFAP is not only expressed by mature astrocytes but also by radial glia during development and neural stem cells in the adult brain, we hypothesized that GFAP expression in astrocytoma might not be a direct indication of glial differentiation and a less malignant phenotype. Therefore, we here review all existing literature from 1972 up to 2018 on GFAP expression in astrocytoma patient material to revisit GFAP as a marker of lower grade, more differentiated astrocytoma. We conclude that GFAP is heterogeneously expressed in astrocytoma, which most likely masks a consistent correlation of GFAP expression to astrocytoma malignancy grade. The GFAP positive cell population contains cells with differences in morphology, function, and differentiation state showing that GFAP is not merely a marker of less malignant and more differentiated astrocytoma. We suggest that discriminating between the GFAP isoforms GFAPδ and GFAPα will improve the accuracy of assessing the differentiation state of astrocytoma in clinical and experimental settings and will benefit glioma classification.

## 1 INTRODUCTION

Gliomas are the most common primary tumors of the central nervous system (CNS). Classically, histological assessment has been used to determine glioma subtype and malignancy grade, which is essential for prognosis and treatment strategies (Wesseling, Kros, & Jeuken, 2011). Since 2016, the World Health Organization (WHO) classification system is applying additional molecular information to improve CNS tumor diagnostics (Louis et al., 2016). This system distinguishes different subtypes of low‐ and of high‐grade glioma based on mutations in isocitrate dehydrogenase 1 (IDH1), and codeletion of the short arm of chromosome 1 and the long arm of chromosome 19 (1q/19p) (Sanson et al., 2009). Despite this new classification, heterogeneity within these subtypes and within individual tumors remains and cell populations with different mutations and expression profiles exist which are likely to have different malignancy characteristics (Patel et al., 2014). Identification of additional molecular characteristics of these subpopulations of cells would greatly benefit diagnostics.

For many years, tissue‐ and cell‐specific expressions of intermediate filament proteins have been useful in tumor diagnostics (Dey, Togra, & Mitra, 2014). In 1971, upon the isolation of filaments from fibrous astrocytes, the 50 kDa type III intermediate filament glial fibrillary acidic protein (GFAP) was identified and characterized. One year later, high expression of GFAP in glioma with astrocyte characteristics, astrocytoma, was described for the first time, followed by many reports thereafter (Delpech et al., 1978; Uyeda, Eng, & Bignami, 1972; van der Meulen, Houthoff, & Ebels, 1978). Multiple studies characterized GFAP expression in glioma subtypes leading to the establishment of GFAP as a biomarker for astrocytoma that is still used to date (Dunbar & Yachnis, 2010). In the healthy human brain, GFAP is mainly expressed in mature astrocytes (Middeldorp & Hol, 2011). Therefore, in clinical as well as fundamental experimental settings, high GFAP expression is believed to mark more differentiated, less malignant tumors. However, more recently GFAP expression was observed in the radial glia of the developing human brain and in adult neural stem cells of the adult brain (Middeldorp & Hol, 2011; Roelofs et al., 2005; van den Berge et al., 2010), showing that GFAP is also expressed in immature, nondifferentiated CNS cells. Since then, GFAP is often used to mark cells with stem cell characteristics in glioma and to target neural stem cells to induce gliomagenesis in animal models (Kwon et al., 2008; J. Chen et al., 2012; Bradshaw et al., 2016; Guichet et al., 2016; Kanabur et al., 2016; Jiang et al., 2017; Welker, Jaros, An, & Beattie, 2017). In addition, GFAP is up‐regulated in non‐neoplastic astrocytes that become reactive in response to the growth of the tumor and do not reflect the differentiation state of neoplastic cells (Gullotta, Schindler, Schmutzler, & Weeks‐Seifert, 1985; Yoshii et al., 1992; H. Y. Yang, Lieska, Glick, Shao, & Pappas, 1993). Therefore, high GFAP levels in tumor specimens may not be a direct indication of a less malignant, more differentiated astrocytoma subtype. Indeed, our recent studies in which we determined the expression of different GFAP isoforms show that higher levels of the alternative splice variant GFAPδ relative to the canonical variant GFAPα are associated with a higher malignant and less differentiated astrocytoma subtype (Stassen et al., 2017). In vitro studies that show a higher malignant gene expression profile and changes in astrocytoma malignant behavior in cells with higher levels of GFAPδ relative to GFAPα, as observed in neurogenic stem cells of the healthy brain (Middeldorp & Hol, 2011; Roelofs et al., 2005; van den Berge et al., 2010), further support the hypothesis of GFAP as a marker of more than lower malignant astrocytoma (Moeton et al., 2014; Stassen et al., 2017).

In order to investigate this hypothesis, we systematically reviewed all existing literature on GFAP expression in patient material of astrocytoma (grade I–IV classified according to the WHO 2007 system or earlier), IDH1 wild‐type (IDHwt) glioma, and IDH1 mutated glioma without a 1q19p codeletion (IDHmut noncodel; classified according to the WHO 2016 system). We included studies that determined the presence of GFAP in control brains and astrocytoma tissue, in astrocytoma of different malignancy grades, in different areas of the tumor, in blood of astrocytoma patients, in proliferating or invasive cells, and studies that describe the morphology of GFAP expressing cells. We conclude that a strong correlation of GFAP to astrocytoma malignancy is absent, that the GFAP positive population is highly heterogeneous, and that distinguishing between the GFAP isoforms GFAPδ and GFAPα might improve the assessment of the differentiation state and malignancy of the tumor and identify subpopulations of GFAP expressing astrocytoma cells.

## 2 SEARCH STRATEGY

Collection of all literature on GFAP expression in astrocytoma patient material was systematically performed. The following code was used to search the PubMed database on August 4, 2015:

“(((((((((((((((((((((((((((((((“Astrocytoma“[Mesh]) OR Astrocytoma[Title/Abstract]) OR astrocytomas[Title/Abstract]) OR glioblastoma[Title/Abstract]) OR glioblastomas[Title/Abstract]) OR astroglioma[Title/Abstract]) OR astrogliomas[Title/Abstract]) OR astrocytic glioma[Title/Abstract]) OR astrocytic gliomas[Title/Abstract]))))) OR astrocytic tumor[Title/Abstract])) OR astrocytic tumors[Title/Abstract])))) OR astrocytic tumor[Title/Abstract]))))) OR gliosarcoma[Title/Abstract])) OR gliosarcomas[Title/Abstract])) OR gliomatosis cerebri[Title/Abstract])) AND ((((((((((((((“Glial Fibrillary Acidic Protein”[Mesh]) OR Glial Fibrillary Acidic Protein[Title/Abstract])) OR Glial Intermediate Filament Protein[Title/Abstract])) OR Glial Intermediate Filament Proteins[Title/Abstract])) OR Glial Fibrillary Acid Protein)) OR astroprotein[Title/Abstract])) OR GFA protein[Title/Abstract])) OR GFAP[Title/Abstract])) NOT (“Animals”[Mesh] NOT (“Animals”[Mesh] AND “Humans”[Mesh]))”.

This resulted in 1322 publications which were scanned on title and abstract by two researchers, independently. Case studies, animal, and in vitro studies were excluded. The 329 remaining studies were evaluated in detail and 77 studies that all analyzed GFAP expression levels or patterns in astrocytoma patient material of grade I, grade II, grade III, or grade IV (WHO classification of the respective year) were finally included. We searched PubMed again with the same search string on September 7, 2018, and an additional 11 papers were included.

## 3 RESULTS

We categorized 88 studies that quantified or described GFAP mRNA or protein expression in human astrocytoma patient material based on the methods that were used and comparisons that were made (Tables 1, 2, 3, 4, 5, 6, 7, 8). The results of the studies in each category are discussed below.

**Table 1:** GFAP expression in astrocytoma compared to control brain tissue

Astrocytoma histological grade	Control tissue	Method	GFAP expression in astrocytoma compared to control	References
Grade IV (*n* = 20)	Whole normal human brain, Non‐tumor gliosis (*n* = 2)	Quantitative immuno‐electrophoresis of tissue extracts	GFAP levels in grade IV tumors are higher compared to normal human brain and nontumor gliosis. Significant effect? *NA*	Dittmann, Axelsen, Norgaard‐Pedersen, & Bock, 1977
Grade III (*n* = 5), Grade IV (*n* = 8)	Adult human and fetal human brain and sheep whole brain extracts	Radial immune‐diffusion of tissue extracts	Grade III: 4.4 U/mg, grade IV: 5.3 U/mg NA, adult human brain: 3 U/mg, Sheep whole brain: 3 U/mg, Fetal human brain: 0.3 U/mg Significant effect? *NA*	Delpech et al., 1978
Grade I (*n* = 1), Grade III (*n* = 13)	Human cortex (*n* = 1)	Radio‐immunoassay of tissue extracts	Increased levels in 1/1 grade I and 6/13 grade III astrocytoma compared to normal cortex. Significant effect? *NA*	Palfreyman, Thomas, Ratcliffe, & Graham, 1979
Grade I (*n* = 1), Juvenile astrocytoma (*n* = 6), Grade III and IV (*n* = 20)	Human cortex (*n* = 1)	2‐DE gel electrophoreses of tissue extracts	Strong increase of GFAP 49 kDa and 49–36 kDa GFAP products in astrocytoma compared to control tissue. Significant effect? *NA*	Narayan, Heydorn, Creed, & Jacobowitz, 1986
Astrocytoma (*n* = 6)	White matter tissue (*n* = 1)	2‐DE gel electrophoreses of tissue extracts	50 kDa GFAP protein was detected in astrocytoma only, 36 kDa spots were detected in both astrocytoma and white matter tissue. Significant effect? *NA*	Luider, Kros, Sillevis Smitt, van den Bent, & Vecht, 1999
Grade I (*n* = 2), Grade II (*n* = 1), Grade III (*n* = 14) Grade IV (*n* = 10)	Epileptic surgery material (*n* = 3, pooled)	2‐DE gel electrophoreses and mass spectrometry of tissue extracts	Increased GFAPα in grade III tumors, decreased GFAPα in grade IV tumors. GFAP_40_ (different mass and isoelectric point; detected peptides within first 270 amino acids) upregulated in both grade III and grade IV. Significant effect? Yes	Chumbalkar et al., 2005
Grade I and II (*n* = 6), Grade III (*n* = 6), Grade IV (*n* = 20)	Healthy brain tissue (taken during removal of meningioma) and peri‐tumor control tissue	Northern blot analysis	Higher GFAP mRNA in 5/7 grade I, 2/5 grade III, 10/18 grade IV astrocytoma compared to healthy brain tissue. GFAP mRNA in four peri‐tumor and tumor pairs: 1/1 grade IV pair increased in tumor; 1/1 grade III pair increased in tumor; 1/1 grade I and II pair increased in tumor. Significant effect? *NA*	Mauro, Bulfone, Turco, & Schiffer, 1991
Grade I (*n* = 1), Grade II (*n* = 5), Grade III (*n* = 6), Grade IV (*n* = 5) All tissues in triplicate	Normal human brain tissue (*n* = 5)	IHC on tissue microarrays (TMA)	Significant higher level of GFAP in astrocytoma compared to normal brain tissue. Significant effect? Yes	Laczko et al., 2007
Low grade (*n* = 9), Grade IV (*n* = 20)	Normal human temporal lobe tissue	IHC on TMAs	GFAP levels increased in grade IV astrocytoma. Significant effect? *NA*	Sharpe & Baskin, 2016
Grade IV (*n* = 64)	Normal human brain tissue (*n* = 10)	qPCR analysis	Increase in grade IV astrocytoma compared to control tissue. The spread in GFAP expression levels was large in tumor tissue. Significant effect? No	Bien‐Moller et al., 2018

NA, not available (authors did not perform statistics); TMA, tissue microarrays; *n*, number of cases.

**Table 2:** GFAP positivity in astrocytoma of different grade using immunohistochemistry

Histological grade	GFAP positive immunostaining	Reference
Grade I (*n* = 6), Grade II (*n* = 6), Grade III (*n* = 21), Grade IV (*n* = 17)	Grade I: 6/6 Grade II: 6/6 Grade III: 21/21 variable amount of GFAP negative cells Grade IV: 4/17 scattered GFAP positive cells	van der Meulen et al., 1978
Low and high grade (*n* = 15)	GFAP positive cells and processes in all astrocytoma.	Chronwall, McKeever, & Kornblith, 1983
Grade I, II, and III (*n* = 58)	54/58: Very high and high expression levels 4/58: Positive but low levels	Gullotta et al., 1985
Grade I (*n* = 9), grade II (*n* = 15), grade III (*n* = 14), grade IV (*n* = 6)	Grade I: 6/9, grade II: 12/15, grade III: 8/14, grade IV: 2/6 Of all positive samples, nine cases showed diffuse, and 18 cases showed partial immunostaining.	Takenaka et al., 1985
Low (*n* = 6) and high (*n* = 6) grade	GFAP positive cells in all astrocytoma.	Yung, Luna, & Borit, 1985
Astrocytoma (*n* = 71)	GFAP reactivity in all tumors.	Herpers, Ramaekers, Aldeweireldt, Moesker, & Slooff, 1986
Low and high grade (*n* = 13)	GFAP reactivity in all tumors.	Royds, Ironside, Taylor, Graham, & Timperley, 1986
Grade I (*n* = 12), grade III (*n* = 9), grade IV (*n* = 24)	GFAP reactivity in all tumors.	Cras, Martin, & Gheuens, 1988
Low and high grade (*n* = 66)	GFAP reactivity in all tumors.	Cruz‐Sanchez et al., 1992
Grade I (*n* = 5), grade 2 (*n* = 5), grade III (*n* = 3), grade IV (*n* = 8)	GFAP reactivity in all tumors.	Zamecnik, Vargova, Homola, Kodet, & Sykova, 2004
Grade III (*n* = 5) and grade IV (*n* = 74)	GFAP reactivity in all tumors.	Hashemi, Naderian, Kadivar, Nilipour, & Gheytanchi, 2014
Grade I, II, and III (*n* = 15), Grade IV (*n* = 10)	GFAP reactivity in all tumors.	Kros, Van Eden, Stefanko, Waayer‐Van, & van der Kwast, 1990
Grade IV (*n* = 10)	GFAP reactivity in all tumors.	Vitolo, Paradiso, Uccini, Ruco, & Baroni, 1996
Grade IV (*n* = 23)	(Focal) GFAP reactivity in all tumors.	Oh & Prayson, 1999
Grade IV (*n* = 82)	Strong GFAP reactivity in 43/82 tumors and weaker in 39/82 tumors.	Donev, Scheithauer, Rodriguez, & Jenkins, 2010
Grade IV (*n* = 39)	GFAP reactivity was almost always observed.	Cuny et al., 2002
Grade IV (*n* = 26)	GFAP reactivity was almost always observed.	Sembritzki, Hagel, Lamszus, Deppert, & Bohn, 2002
Grade IV (*n* = 30)	GFAP reactivity in all tumors.	Terada, 2015
Low grade (*n* = 40) , grade IV (*n* = 16)	GFAP reactivity in all tumors.	Goyal et al., 2015
Grade II ((*n* = 43), grade III and IV (*n* = 33)	Only report decreasing GFAP levels with increasing grade.	Xing et al., 2016

*n*, number of cases.

**Table 3:** GFAP positive cell quantification in astrocytoma of different grades using immunohistochemistry (no statistics)

Histological grade	Scoring system	GFAP positive cell score	Reference
Grade III (*n* = 1), Grade IV (*n* = 11)	0, 5–25%, 25–50%, 75–100%	0: ‐ 5–25%: 2 grade IV 25–50%: 4 grade IV 75–100%: 5 grade IV, 1 grade III	Jones, Bigner, Schold Jr.,Eng, & Bigner, 1981
Grade IV (*n* = 97)	Not present, In single cells, In groups of cells, In more than 30% of cells	Not present: 1/97, Single cells: 16/97, Groups of cells: 20/97, More than 30% of cells: 60/97	Schmidt et al., 2002
Grade II (*n* = 5), Grade III (*n* = 10), Grade IV (*n* = 26)	Negative, single cells, cluster of cells (20–50%), 50%–90% of cells are positive, Almost 100% are positive	Grade I and II: >50% of the cells Grade III and IV: Single cells or negative	Peraud et al., 2003
Grade I and II (*n* = 5), Grade IV (*n* = 4)	Scores from 0 to 3	9/9 score 2 or 3	Tan, Magdalene Koh, & Tan, 2006
Grade II (*n* = 10), Grade III (*n* = 11), Grade IV (*n* = 5)	Percentage of positive cells	All astrocytoma examined ranged from 5% to 100% of positive cells.	Rousseau et al., 2006
Grade I (*n* = 8), Grade IV (*n* = 8)	No positive cells, 1–10% of positive cells, 11–25%, 26–50%, 51–90%, 91–100%	Grade I: 8/8 26–50% Grade IV: 8/8 51–100%	Colin et al., 2007
Grade I (*n* = 15), Grade II (*n* = 26), Grade III (*n* = 4), Grade IV (*n* = 8)	0, 10%, 11–50%, 50–75%, >75% of positive cells Weak, medium, strong intensity	Grade I: 15/15 > 75% Grade II: 24/26 > 75%, 2/26 50–75% Grade III: 4/4 > 75% Grade IV: 7/8 75%, 1/8 50–75% GFAP intensity was strong in all cases.	Shuangshoti et al., 2009
Grade II (*n* = 25), Grade III (*n* = 29), Grade IV (*n* = 41)	<5%, >5%	Grade II: 25/25 > 5% Grade III: 26/29 > 5% Grade IV: 39/41 > 5%	Liu, Lu, Ohgaki, Merlo, & Shen, 2009

Statistics were not performed or mentioned in these studies. *n*, number of cases.

**Table 4:** GFAP positive cell quantification in astrocytoma of different grades using immunohistochemistry (statistics)

Histological grade	Scoring system	GFAP positive cell score	Significant effect?	Reference
Grade I (*n* = 9), Grade II (*n* = 8), Grade III (*n* = 7), Grade IV (*n* = 14)	0, <1%, <5%, <10%, <25%, <50%, <95%, 95–100%	Separation of stromal and neoplastic cells (morphology nucleus): Stromal cells in tumor: 36/38 Neoplastic cells: 37/38 (mostly 25%; bell‐shaped distribution)	No	Bishop & de la Monte, 1989
Grade I and II (*n* = 10), Grade III and IV (*n* = 29)	<25%, 25–50%, 50–80%	Positive staining found in all tumors. No difference between grades.	No	Nakopoulou, Kerezoudi, Thomaides, & Litsios, 1990
Grade I and II (*n* = 16), Grade III (*n* = 15), Grade IV (*n* = 21)	0–25%, 25–50%, 50–75%, >75%	Tumors with the lowest number of GFAP positive cells were observed in grade III and IV.	No	Kajiwara et al., 1992
Grade I (*n* = 6), Grade II (*n* = 12), Grade III (*n* = 12), Grade IV (*n* = 12)	Automated measurement of specific staining intensity. Primary and recurrent tumor pairs.	No difference between malignancy grades, or recurrent versus primary tumors.	No	Stan et al., 1999
Grade I (*n* = 7), Grade II (*n* = 13), Grade III (*n* = 7), Grade IV (*n* = 23)	1: <5% 2: 1–5% 3: >25%	No significant difference between grade I and II and grade III and IV tumors.	No	Ikota, Kinjo, Yokoo, & Nakazato, 2006
Grade II (*n* = 9), Grade III (*n* = 48), Grade IV (*n* = 70)	Automatic counting and stratification into negative, low intensity, high intensity	All gliomas were pooled together: No differences between histological grades. (Not tested within astrocytoma only.)	No	Cheung, Corley, Fuller, McCutcheon, & Cote, 2006
Grade I and II (*n* = 42), Grade III and IV (*n* = 47)	<25%, 26–50%, 51–75%, 76–100%	Average GFAP cell percentage: Grade I and II: 65.4 Grade III and IV: 52.9	Yes, *p* = 0.027	Hlobilkova et al., 2007
Grade I (*n* = 1), Grade II (*n* = 5), Grade III (*n* = 6), Grade IV (*n* = 5)	Score 1–5 1: <20% 5: >80% (TMA; automated counting)	Decreasing GFAP level scores with increasing astrocytoma grade.	Yes, *p* < 0.05	Laczko et al., 2007
Grade I (*n* = 7), Grade II (*n* = 14), Grade III (*n* = 15), Grade IV (*n* = 17)	% of GFAP positive cells (counting of positive vs. total amount of cells)	Higher % of GFAP positive cells in grade I and II compared to III and IV.	Yes, *p* = 0.00	L. Yang et al., 2014
Low‐ and high‐grade astrocytoma (*n* = 50)	0: No staining; 1: Rare or <1% positive cells; 2: 2–30% positive cells; 3: 31–60% positive cells; 4: 61% positive cells (TMA)	Average scores: Grade I: 3.67 Grade II: 2.97 Grade III: 3.57 Grade IV: 3.12	No	Rushing, Sandberg, & Horkayne‐Szakaly, 2010
Grade I (*n* = 94 primary, 19 recurrences), Grade II (*n* = 71 primary, 10 recurrences), Grade III (*n* = 62 primary, 16 recurrences), Grade IV (*n* = 120 primary, 28 recurrences)	Quantity: 0: <1% 1: 1–24% 2: 25–49% 3: 50–74% 4: 75–100% Intensity: 0: No staining 1: Weak staining, 2: Moderate staining 3: Strong staining Staining score: Quantity*intensity (0–12 range)	Correlation of GFAP to malignancy grade. Grade I higher levels compared to other grades. Grade II significantly higher compared to grade IV. Within grade II glioma GFAP levels were higher in: IDHmut compared to IDHwt ATRX loss compared to ATRX retention No significant correlation of GFAP to survival after correction for age and tumor grade.	Yes, *p* < 0.0001 No Yes, *p* = 0.0022 Yes, *p* = 0.0033 Yes, *p* = 0.0214	Schwab et al., 2018

TMA, tissue microarrays; IDHwt, IDH1 wild‐type; IDHmut, IDH1 mutated; ATRX, alpha‐thalassemia/mental retardation syndrome X‐linked; *n* = number of cases.

**Table 5:** GFAP quantification in astrocytoma homogenates

Histological grade	Method	GFAP expression levels	Significant effect?^a^	Reference
Protein				
Grade II (*n* = 3), Grade III (*n* = 1), Grade IV (*n* = 7)	Rocket immuno‐electrophoresis	Grade II: >12 μg/mg Grade III: 47.5 μg/mg (*n* = 1) Grade IV: <2 μg/mg	NA	Jacque et al., 1978
Grade III (*n* = 5), Grade IV (*n* = 8)	Radial immunodiffusion	No difference between grades.	No	Delpech et al., 1978
Low grade (*n* = 4), Grade IV (*n* = 6)	Quantitative immune‐electrophoresis	Variation in levels, no correlation to malignancy grade.	No	Rasmussen, Bock, Warecka, & Althage, 1980
One sample of each grade	Western blot (to confirm staining data)	Decreased GFAP levels in grade III and IV compared to grade I and II.	NA	Peraud et al., 2003
Grade II (*n* = 10), Grade IV (*n* = 10)	2‐DE gel electrophoreses and mass spectrometry	Higher levels of GFAP in grade II compared to grade IV extracts. GFAP was one of the 15 proteins that was differentially expressed.	Yes	Odreman et al., 2005
RNA				
Grade I and II (*n* = 6), Grade III (*n* = 6), Grade IV (*n* = 20)	Northern blot analysis, 2,600 bp probe	No difference between astrocytoma grades.	No	Mauro et al., 1991
Low grade (*n* = 7), Grade III (*n* = 9), Grade IV (*n* = 14)	Northern blot analysis	No difference between astrocytoma grades.	NA	Dmytrenko et al., 2009
Grade II (*n* = 55), Grade III (*n* = 105), Grade IV (*n* = 150)	RNA sequencing data; normalized gene expression values (the cancer genome atlas)	Higher levels in grade II and III astrocytoma compared to grade IV.	Grade II versus IV: FDR = 1.58E−10 Grade III versus IV: FDR = 3.92E−7	Stassen et al., 2017

FDR, false discovery rate; NA, not available (authors did not perform statistics); *n*, number of cases.

*p* values are given if provided.

**Table 6:** GFAP staining patterns in astrocytoma of different grade

Histological type	Described patterns (1–15)	Described differences between astrocytoma grades	Reference
Grade III (*n* = 5), Grade IV (*n* = 8)	1. Immunofluorescence found in the cell body and processes of malignant cells. 7. Peri‐vascular staining. 8. GFAP expression aligning the inner surface of the plasma membrane.	–	Delpech et al., 1978
Astrocytoma (*n* = 4), Grade IV (*n* = 8)	1. Immunofluorescence found in the cell body and processes of malignant cells. 8. GFAP expression aligning the inner surface of the plasma membrane. 9. High GFAP expressing gemistocytic cells. 10. Uniform GFAP expression with extensions into short processes. 11. High GFAP expression in stellate‐shaped elongated, bipolar, piloid cells.	–	Duffy, Huang, Rapport, & Graf, 1980
Low grade (*n* = 17), Grade III (*n* = 16), Grade IV (*n* = 7)	1. Immunofluorescence found in the cell body and processes of malignant cells. 2. Dense meshwork of GFAP positive cellular processes, stained over their full Length, including thin processes. 3. GFAP negative microcystic (small cell) areas. 10. Uniform GFAP expression with extension into short processes.	3. More often observed in higher grade astrocytoma	Velasco, Dahl, Roessmann, & Gambetti, 1980
Grade I (*n* = 5), Grade II and III (*n* = 27), Grade IV (*n* = 10)	1. Immunofluorescence found in the cell body and processes of malignant cells. 3. GFAP negative microcystic (small cell) areas. 9. High GFAP expressing gemistocytic cells. 13. GFAP expression in multinucleated cells. 15. Necrotic GFAP negative areas.	13. Observed in grade IV astrocytoma 15. Observed in grade IV astrocytoma	Tascos, Parr, & Gonatas, 1982
Low and high grade (*n* = 15)	7. Peri‐vascular staining.	–	Chronwall et al., 1983
Grade I, II, and III (*n* = 58)	3. GFAP negative microcystic (small cell) areas.	3. More often observed in higher grade astrocytoma	Gullotta et al., 1985
Astrocytoma (*n* = 15)	1. Immunofluorescence found in the cell body and processes of malignant cells. 3. GFAP negative microcystic (small cell) areas. 11. High GFAP expression in stellate‐shaped elongated, bipolar, piloid cells. 12. GFAP expressing cells arranged in parallel bundles. 13. GFAP expression in multinucleated cells. 14. GFAP expression in the rim of Rosenthal fibers.	–	Smith & Lantos, 1985
Low and high grade (*n* = 13)	6. Diffuse/homogeneous GFAP staining	6. More often in low‐grade astrocytoma	Royds et al., 1986
Astrocytoma (*n* = 71)	2. Dense meshwork of GFAP positive cellular processes, stained over their full length, including thin processes. 7. Peri‐vascular staining.		Herpers et al., 1986
Grade I (*n* = 12), Grade III (*n* = 9), Grade IV (*n* = 24),	1. Immunofluorescence found in the cell body and processes of malignant cells. 4. Focal GFAP expression only. 7. Peri‐vascular staining.	4. More often seen in grade III and specifically grade IV astrocytoma	Cras et al., 1988
Low and high grade (*n* = 66)	4. Focal GFAP expression only. 6. Diffuse/homogeneous GFAP staining.	4. More often seen in high astrocytoma. 6. More often in low‐grade astrocytoma.	Cruz‐Sanchez et al., 1992
Grade IV (*n* = 23)	4. Focal GFAP expression only.		Oh & Prayson, 1999
Grade I (*n* = 8), Grade II (*n* = 18), Grade III (*n* = 4), Grade IV (*n* = 30)	6. Diffuse/homogeneous GFAP staining.	6. More often seen in grade IV compared to grade I and II	Katsetos et al., 2001
Grade IV (*n* = 39)	1. Immunofluorescence found in the cell body and processes of malignant cells. 5. GFAP positive large‐sized cells with numerous processes but variable staining Intensity.		Cuny et al., 2002
Grade II (*n* = 5), Grade III (*n* = 10), Grade IV, (*n* = 26)	1. Immunofluorescence found in the cell body and processes of malignant cells. 9. High GFAP expressing gemistocytic cells.	1. Staining of processes more often observed in low‐grade astrocytoma	Peraud et al., 2003
Grade I (*n* = 5), Grade II (*n* = 5), Grade III (*n* = 3), Grade IV (*n* = 8)	2. Dense meshwork of GFAP positive cellular processes, stained over their full length, including thin processes.	2. Dense fibrillary network mainly in grade I and II astrocytoma 2. Shorter processes in grade III and IV astrocytoma	Zamecnik et al., 2004
Grade I (*n* = 37), Grade II (*n* = 11)	2. Dense meshwork of GFAP positive cellular processes, stained over their full length, including thin processes. 3. GFAP negative microcystic (small cell) areas.		Tanaka, Sasaki, Ishiuchi, & Nakazato, 2008

*n* = number of cases.

**Table 7:** GFAP isoform expression in astrocytoma

Histological type	Methods	GFAP isoform expression	Reference
Grade III (*n* = 8), Grade IV (*n* = 1), Frontal cortex control (*n* = 1)	Quantitative PCR	GFAPα, GFAPδ, and GFAPκ expression is decreased in 5/8 and increased in 3/8 astrocytoma compared to control tissue. The GFAPκ/GFAPδ ratio is increased in all astrocytoma compared to control tissue.	Blechingberg et al., 2007
Grade I (*n* = 4), Grade IV (*n* = 2), Control healthy and epileptic tissue	Immunohistochemistry; No quantification	GFAPδ is coexpressed with GFAP in reactive astrocytes. GFAPδ expressed in 1/4 grade I astrocytoma, focal expression in 3/4 grade I tumors, but mostly negative. Grade IV: Strong focal GFAPδ expression	Andreiuolo et al., 2009
Grade I (*n* = 3), Grade II (*n* = 5), Grade III (*n* = 4), Grade IV (*n* = 4), Control autopsy material (*n* = 4)	Immunohistochemistry; Quantification of mean grey value after outlining the cell	Increased pan GFAP and GFAPδ expression in astrocytoma compared to control tissue. Pan GFAP levels significantly increase from grade I to grade III and are decreased in grade IV astrocytoma. GFAPδ expression is mostly undetectable in control cells and increases with increasing astrocytoma grade. GFAP positive control cells are stellate‐shaped with well‐developed processes. Grade I and II: Stellate polygonal or round cells Grade III and IV: Round and spindle shaped cells GFAPδ is mainly observed in cell bodies not in processes. Inverse correlation of GFAPδ expression intensity and the amount of processes (round>polygonal>stellate).	Choi, Kwak, Kim, Sheen, & Kang, 2009 (same lab as Heo et al., 2012)
Spinal cord astrocytoma Grade I (*n* = 3), Grade II (*n* = 14), Grade III (*n* = 5)	Immunohistochemistry; Quantification of mean grey value after outlining the cell	Increased GFAPδ expression in spinal cord astrocytoma compared to control tissue. Strong positive correlation of GFAPδ with astrocytoma grade. Weaker positive correlation of pan GFAP with astrocytoma grade. Grade I and II: GFAPδ expression in stellate polygonal or round cells. Grade III: GFAPδ expression in round and spindle‐shaped cells.	Heo et al., 2012 (same lab as Choi et al., 2009)
Grade II (*n* = 7), Grade III (*n* = 2), Grade IV (*n* = 35)	Immunohistochemistry; Quantification of GFAPδ positive cell number Analysis of MRI scans: High invasive: Corpus callosum infiltration with opposite hemisphere invasion and multicentric astrocytoma with tumor foci in both hemispheres Low invasive: Unifocal deep‐seated astrocytoma	7/7, 2/2 and 34/35 astrocytoma express GFAPδ Significant increase in GFAPδ expression in grade IV (83.8%) compared to grade II (57.1%). Significant correlation of GFAPδ to neuroimaging invasiveness. Two low‐grade invasive tumors show high levels of GFAPδ.	Brehar, Arsene, Brinduse, & Gorgan, 2014
Grade II (*n* = 55), Grade III (*n* = 105), Grade IV (*n* = 150)	RNA sequencing data; normalized isoform expression values (the cancer genome atlas)	Significant decrease in the level of GFAPα in grade IV astrocytoma and a significant increase in the relative level of GFAPδ to GFAPα in grade IV astrocytoma.	Stassen et al., 2017

*n* = number of cases.

**Table 8:** GFAP levels in body fluids of astrocytoma patients

Histological grade	Method	Results	Significant effect?^a^	Reference
Low grade (*n* = 3), Grade IV (*n* = 2)	GFAP levels in cerebrospinal fluid (CSF) of patients by radiolabeling of GFAP with Iodine‐125	High levels of GFAP in CSF can discriminate between astrocytoma and other types of tumors and controls.	NA	Szymas, 1985
Grade II (*n* = 13), Grade III (*n* = 14), Grade IV (*n* = 50), Healthy controls (*n* = 50)	GFAP levels in serum determined by ELISA	Detected in 40/50 grade IV astrocytoma. Higher levels compared to other tumors and controls. Correlation of GBM patient serum GFAP with: Tumor volume, Tumor necrosis volume, and Number of necrotic GFAP positive cells.	Yes, *p* < 0.0001 Yes, *p* < 0.0001 Yes, *p* = 0.004 Yes, *p* = 0.007	Jung et al., 2007
Grade IV (*n* = 61), Healthy controls	GFAP positive circulating microparticles (MP) in blood detected by flow cytometry before and after surgery	Baseline: Baseline MP level higher in grade IV versus control Increase in GFAP positive MPs after 7 days that persist up to 7 months after all surgeries (1 month and 4 months measurements as well). Higher number of GFAP positive MPs at 7 months compared to 7 days for subtotal resections only. Increased GFAP positive MPs after 7 months compared to 4 months in patients with radiological disease progression.	Yes Yes, *p* = 0.05 Yes, *p* < 0.05 Yes, *p* = 0.026	Sartori et al., 2013
Grade II (*n* = 7), Grade III (*n* = 10), Grade IV (*n* = 34), Intercranial metastasis (ICM) (*n* = 41), MS patients (*n* = 25), Healthy controls (*n* = 26)	GFAP levels in serum determined by ELISA	GFAP detected in 0/7 grade II, 0/10 grade III, 13/34 grade IV, 1/41 metastasis, 1/25 MS patients, and 1/26 healthy controls. Significant association with grade IV astrocytoma diagnosis. Higher serum levels in grade IV compared to ICM. Grade IV: No correlation to neuroradiological characteristics Patients with detectable GFAP in serum levels had a longer survival probability (11.3 months) compared to others (5.2 months).	Yes, *p* < 0.001 Yes, *p* < 0.05 No No, *p* = 0.18	Ilhan‐Mutlu et al., 2013
Grade IV (*n* = 141), Noncancer controls (*n* = 23), Brain metastasis (*n* = 5)	Isolation of mono‐nucleated cells from peripheral blood and immunocytochemistry of cells	GFAP positive cells were detected in: 29/141 grade IV, 0/23 healthy controls, 1/5 brain metastasis GFAP positive cells were more often detected in patients with EGFRvIII mutated tumors. There was no correlation to survival.	Yes Yes, *p* = 0.04 NA	Muller et al., 2014
Grade IV (*n* = 111), healthy controls (*n* = 99), nonglial brain tumors (*n* = 40)	GFAP levels in serum determined by ELISA	Higher levels in grade IV compared to healthy controls. Higher levels in grade IV compared to nonglial tumors. Detected in 30/111 patients. Larger tumor volume in patients with detectable compared to undetectable GFAP levels. No correlation to PFS or survival.	Yes, *p* < 0.01 Yes, *p* = 0.04 Yes, *p* < 0.001 *p* = 0.11, *p* = 0.48	Gállego Pérez‐Larraya et al., 2014
Grade II (*n* = 11), Grade IV (*n* = 23), Healthy controls (*n* = 15)	GFAP levels in serum determined by ELISA	Above detection level in 2/15 healthy controls, 3/11 grade II, and 9/23 grade IV astrocytoma patients. Higher levels in grade IV patients.	No	Lange et al., 2014
Grade I (*n* = 3), Grade II (*n* = 5), Grade III (*n* = 3), Grade IV (*n* = 25), Brain metastasis (*n* = 24), Healthy individuals (*n* = 132)	GFAP levels in serum determined by ELISA and immunohistochemistry of tumor tissue.	Higher GFAP levels in grade IV glioma compared to all others. Higher level of GFAP expression in the tumor subgroup with high GFAP serum levels.	*p* < 0.001 *p* = 0.035	Tichy et al., 2016
Recurrent tumors: Grade IV IDHmut (*n* = 4), Grade IV IDHwt (*n* = 3), Grade III (*n* = 3), Healthy controls (*n* = 3)	GFAP positive CD9+ exosomes in serum of patients determined by flow cytometry	GFAP positive CD9+ exosomes of total CD9+ exosomes were 7.9 times higher in patients with recurrent glioma (22.8%; 18.2–27.1%) compared to healthy controls (2.9%; 2.7–3.2%) at baseline.	NA	Galbo et al., 2017
Low grade (*n* = 2), Grade IV IDHwt (*n* = 29), Grade IV IDHmut (*n* = 1), Unknown (*n* = 3)	GFAP levels in serum determined by a sandwich immunoassay	14 of 33 grade IV glioma patients showed high GFAP levels prior to surgery. 6 weeks after surgery GFAP levels in GFAP positive patients were decreased compared to levels prior to surgery. No increase in any of the patients. No correlation with tumor volume, survival or PFS.	NA	Vietheer et al., 2017
Grade IV (*n* = 25), Brain metastasis (*n* = 7)	GFAP levels in serum determined by ELISA	Increase in GFAP levels directly after surgery. No further increase up to 7 days after surgery. Trend for increase after surgery for brain metastasis also observed. Higher levels in grade IV glioma compared to brain metastasis prior to surgery. No correlation to tumor volume.	*p* = 0.0172 *p* = 0.0198	Baumgarten et al., 2018
Grade III (*n* = 13), Grade IV (*n* = 14), Healthy controls (*n* = 13)	GFAP levels in serum determined by ELISA	Preoperative: Higher levels of GFAP in grade IV compared to grade III. Higher levels of GFAP in grade IV compared to controls. Correlation to enhancing tumor volume (primary/recurrence). Correlation to necrotic tumor volume (primary/recurrence). Lower levels in IDHmut glioma. Correlation to Ki67 expression. No correlation of GFAP expression in tumor to serum levels. High GFAP levels related to poor PFS in primary tumors. Postoperative: Increased GFAP levels in 65% of glioma patients. No correlation to tumor volume. No difference between day 2, 3, 4, and 5 postsurgery.	*p* = 0.003 *p* = 0.001 *p* = 0.005/0.011 *p* = 0.001/0.047 *p* = 0.016 *p* < 0.001 *p* = 0.008 *p* = 0.003	Kiviniemi et al., 2015

NA, not available (authors did not perform statistics); PFS, progression free survival; IDHwt, IDH1 wild‐type; IDHmut, IDH1 mutated; *n,* number of cases; EGFRvIII, epidermal growth factor receptor gene amplification.

*p* values are given if provided.

### 3.1 GFAP expression is increased in astrocytoma versus healthy human CNS tissue

Two early studies already address one of the most important questions considering GFAP in astrocytoma; is the GFAP protein in astrocytoma different from GFAP in the healthy brain, in respect of its immunochemical characteristics and expression level? Delpech et al. (1978) used radial immunodiffusion analysis to demonstrate that GFAP in healthy human brain extracts is immunochemically similar to GFAP in astrocytoma extracts (Delpech et al., 1978). This agrees with the study of Dittmann et al. (1977) who show, by rocket immunoelectrophoresis, that there are no immunochemical differences between GFAP isolated from astrocytoma and the adult human brain. However, GFAP isolated from fetal human brain has different characteristics in this assay when compared to GFAP from astrocytoma or adult human brain (Dittmann et al., 1977). Both studies, and many studies that followed hereafter, show that GFAP expression in astrocytoma is increased compared to healthy brain tissue (Table 1) both at the protein (Delpech et al., 1978; Dittmann et al., 1977; Mauro et al., 1991; Narayan et al., 1986; Palfreyman et al., 1979) and mRNA (Bien‐Moller et al., 2018; Laczko et al., 2007) level. In contrast, a more recent study that used two‐dimensional (2‐DE) gel electrophoresis followed by mass spectrometry, shows a decrease in the 50 kDa canonical GFAP protein in grade IV astrocytoma (although this protein is increased in grade III astrocytoma; Chumbalkar et al., 2005). Interestingly, a second GFAP protein of different mass and isoelectric point was detected in this study, which is upregulated in both grade III and grade IV astrocytoma. This suggests the expression of different GFAP proteins in these tumors, which potentially are isoforms, differently phosphorylated proteins, or degradation products. A variety of GFAP proteins with different molecular weights are described in a second 2‐DE gel electrophoresis study that compares astrocytoma to healthy brain tissue, and the results show a higher expression of a 49 kDa GFAP protein and proteins ranging from 36 to 49 kDa in astrocytoma (Narayan et al., 1986). In a third study, 36 kDa and 50 kDa GFAP proteins are detected in astrocytoma, whereas only the 36 kDa GFAP protein is detected in control white matter tissue (Luider et al., 1999). Together, these studies show that the canonical GFAP protein is increased in astrocytoma compared to healthy brain tissue, but also that the detection of different GFAP forms (either splice isoforms, differentially phosphorylated forms, or degradation products) can be potentially used to discriminate between astrocytoma and healthy tissue, and astrocytoma of different malignancy grades. For example, lower molecular weight GFAP fragments that result from caspase‐mediated proteolysis are indicative of cellular stress and could be used as an additional biomarker for astrocytoma. These lower molecular weight GFAP fragments have been detected in brain extracts of Alexander disease patients, a rare nervous system disorder caused by GFAP mutations (M.‐H. Chen, Hagemann, Quinlan, Messing, & Perng, 2013; Lin, Messing, & Perng, 2017). As specific antibodies that detect these fragments are available (M.‐H. Chen et al., 2013; Lin et al., 2017), it might be interesting to use these to check for the presences of the caspase‐cleaved fragments in astrocytoma as well.

### 3.2 Inconsistent correlation of GFAP expression levels to astrocytoma malignancy

In Tables 2, 3, 4, we show all published results on general GFAP expression levels in astrocytoma grade I, II, III, and IV as determined by immunohistochemistry. Table 2 lists all studies that classified tumors of different grades as positive or negative for GFAP, without any form of quantification. Seventeen out of 20 studies show GFAP expression in all astrocytomas, although three of these 17 studies describe a focal or weaker expression in some tumors (Donev et al., 2010; Gullotta et al., 1985; Oh & Prayson, 1999). Only two out of the 20 studies specify the malignancy grade in which GFAP negative areas or tumors are found. Takenaka et al. (1985) describe GFAP negative tumors in astrocytoma of all grades (Takenaka et al., 1985). van der Meulen et al. (1978) describe GFAP immunoreactivity in all grade I and II astrocytomas, the presence of GFAP negative areas in grade III, and show that four out of 17 grade IV astrocytomas are GFAP negative. Quantification of differences in GFAP expression between astrocytoma grades was not performed in the studies listed in Table 2. Nevertheless, the observation of a decreasing number of GFAP positive cells with increasing astrocytoma grade is frequently mentioned (Cras et al., 1988; Cruz‐Sanchez et al., 1992; Gullotta et al., 1985; Oh & Prayson, 1999; Royds et al., 1986; van der Meulen et al., 1978; Xing et al., 2016). One of the studies, however, describes stronger staining of GFAP in grade III compared to lower grade astrocytoma (Cruz‐Sanchez et al., 1992).

Studies in which a scoring system is used to quantify the level of GFAP immunostaining are listed in Tables 3 and 4. In Table 3, data of studies are shown in which GFAP is quantified without applying statistics to test for the significance of differences observed between astrocytomas. In six out of eight of these studies, there are no clear differences between GFAP scores in low‐ and high‐grade astrocytoma. One study reports lower GFAP immunostaining scores in grade III and IV compared to I and II (Peraud et al., 2003) and in contrast, another study describes higher GFAP scores in grade IV compared to grade I astrocytoma (Colin et al., 2007). In the studies listed in Table 4, statistical testing is used to determine the significance of the differences in GFAP immunostainings between astrocytoma of different grades. Two out of 11 studies that compare grade I and II to grade III and grade IV, show significant higher GFAP scores in grade I and II astrocytoma (Hlobilkova et al., 2007; L. Yang et al., 2014). In one study, a significant decrease in GFAP levels with increasing astrocytoma grade is reported (Laczko et al., 2007) and a fourth study finds a significant correlation of GFAP levels to malignancy grade and a significant difference between grade II and IV astrocytoma (Schwab et al., 2018). Seven out of the 11 studies failed to show significant differences in the percentage of GFAP positive cells between astrocytoma of different grades of malignancy. Based on these studies that used immunohistochemistry analyses, we conclude that GFAP protein levels or the number of GFAP positive cells do not correlate to astrocytoma malignancy grade.

Within grade IV astrocytoma, GFAP has been associated with specific grade IV subtypes which are determined based on either the expression pattern of different intermediate filament proteins (Skalli et al., 2013) or the expression of different proteins including GFAP (Motomura et al., 2012). There is no significant difference in the survival probability of patients with either of the grade IV subtypes that were identified based on intermediate filament protein expression (GFAP, vimentin, nestin, and synemin; Skalli et al., 2013). However, patients with the grade IV subtype that correlates with high GFAP expression as identified by Motomura et al. (2012), the astrocytic mesenchymal subtype, have significantly worse survival probability compared to the oligodendrocyte precursor subtype with low GFAP expression (Motomura et al., 2012). These results further emphasize the absence of a consistent correlation of GFAP expression to a more differentiated, lower malignant astrocytoma.

Table 5 lists the studies that quantified GFAP expression levels in astrocytoma homogenates. Four out of eight studies show no significant differences in GFAP protein or RNA levels between the different astrocytoma grades. Two studies that quantified RNA (Dmytrenko et al., 2009) or protein levels (Rasmussen et al., 1980) both report a high variability in expression. In two other studies that quantified protein levels, GFAP is higher in grade II compared to grade IV astrocytoma (Jacque et al., 1978; Odreman et al., 2005), which reached statistical significance when tested (Odreman et al., 2005). In addition, a third study reports higher GFAP levels in grade I and II compared to grade III and IV astrocytoma but only show western blot evidence for one sample of each grade (Peraud et al., 2003). In short, studies that analyzed astrocytoma homogenates neither generate consistent results on the correlation of GFAP expression to astrocytoma malignancy grades and additional analyses in larger cohorts are needed. In our own previously published study in which we used RNA sequencing data of 310 patients available at TCGA we do show a strong decrease in general GFAP mRNA levels in grade IV compared to grade II and grade III astrocytoma (Stassen et al., 2017).

### 3.3 GFAP is heterogeneously expressed in astrocytoma

The above‐described studies show a large variation in outcomes. To a certain extent, variation between studies is explained by the different methods of analysis, grouping of patient samples, sample sizes, scoring systems, antibodies used, quality of tissues, and variation in staining intensity within and between tumors (Cuny et al., 2002). Moreover, the complexity of GFAP positive cell morphology and staining patterns hamper quantification of immunohistochemistry data of the number of GFAP positive cells to the total number of cells (Tanaka et al., 2008).

Besides these methodological issues, the inconsistent correlation of GFAP expression to astrocytoma malignancy might results from heterogeneity in localization and function of GFAP positive astrocytoma cells within and between tumors. Local variability of GFAP immunostaining was highlighted in many studies (Cras et al., 1988; Cruz‐Sanchez et al., 1992; Gullotta et al., 1985; Herpers et al., 1986; Royds et al., 1986; Sharpe & Baskin, 2016; Tascos et al., 1982; van der Meulen et al., 1978; Velasco et al., 1980). Thus, as was already noted in 1985 (Gullotta et al., 1985) and many times thereafter (Hashemi et al., 2014; Sembritzki et al., 2002) analysis of different areas of the same tumor is necessary to determine GFAP expression levels of a tumor. This is emphasized by two studies that specifically analyzed regional differences within astrocytoma grade IV. Nagashima, Suzuki, Asai, and Fujimoto (2002) separated grade IV astrocytoma in two areas: core and periphery. They describe the presence of GFAP positive cells in both the periphery and the core. Pistollato et al. (2010) confirmed these findings and studied cell number and characteristics of the regions in more detail. Grade IV tumors were subdivided into a necrotic and hypoxic core, a transitional intermediate area, and a hypervascularized periphery. They describe that the highest number of GFAP expressing cells is present in the periphery of the tumor surrounding endothelial cells. GFAP expression significantly decreases toward the tumor core but is still present in a few individual cells (Pistollato et al., 2010). These local differences in the number of GFAP expressing cells complicate the quantification of GFAP levels of grade IV astrocytoma. GFAP negative areas are mostly seen in grade III and grade IV astrocytoma (Gullotta et al., 1985; Tascos et al., 1982; Velasco et al., 1980), resulting in unreliable quantification of total GFAP levels using small areas of specifically these tumors.

Many studies have described large heterogeneity in immunostaining characteristics and the morphology of GFAP positive cells (Table 6). In general, GFAP immunofluorescence is found in the cell body and processes of neoplastic cells and these immunostainings clearly visualize cell morphology. More specifically, GFAP is expressed in gemistocytic cells with uniform staining in cell bodies with shorter processes, bipolar elongated cells, cells arranged in parallel bundles, stellate/piloid cells, multinucleated cells, cells associated with the vasculature, and cells expressing GFAP aligning the inner surface of the plasma membrane only. GFAP positive immunostaining ranges from a dense meshwork of GFAP positive processes without a clear visible cell body to areas with single large gemistocytic cells with strong cytoplasmic staining and short processes. Microcystic areas within the tumor are described to be negative for GFAP, although weak GFAP staining in these areas has been described as well. While these characteristics are observed in all astrocytoma grades, some have been observed more frequently in low‐ or in high‐grade astrocytoma. GFAP negative microcystic (Gullotta et al., 1985; Velasco et al., 1980) and necrotic areas (Tascos et al., 1982), and focal GFAP staining are more often seen in higher grade astrocytoma (Cras et al., 1988; Cruz‐Sanchez et al., 1992), whereas diffuse GFAP staining (Cruz‐Sanchez et al., 1992; Royds et al., 1986) and a dense fibrillary network with clear staining of processes (Peraud et al., 2003; Zamecnik et al., 2004) are more often seen in lower grade astrocytoma with one study specifically reporting on shorter processes in grade III and grade IV astrocytoma (Zamecnik et al., 2004). However, as GFAP positive neoplastic cells are intermingled with GFAP positive reactive astrocytes, determining the neoplastic origin of these thin and complex processes is complicated (Gullotta et al., 1985). Although most studies report to be able to discriminate between these two cell types, and distinctions in the morphology of reactive and neoplastic astrocytes have been clearly described (Yoshii et al., 1992), quantification of process‐rich GFAP positive areas might lead to an overestimation of GFAP levels in neoplastic cells. Additional immunohistochemistry of for example the 300 kDa intermediate filament associated protein (IFAP) specifically expressed in neoplastic astrocytes (H. Y. Yang et al., 1993), and the more recently identified ATRX, that is lost in neoplastic but present in reactive astrocytes (Mellai et al., 2017), might be helpful in distinguishing these cell types.

In accordance with the variability in cell morphology and immunostaining characteristics that imply functional heterogeneity of GFAP positive astrocytoma cells, GFAP is expressed in cells with different biological functions that contribute to tumor malignancy. The degree of proliferation and invasion are the two most important traits of astrocytoma malignancy, and we examined the publications that describe the level of GFAP expression in proliferating and invading cells in human astrocytoma material. Two studies report absence of GFAP expression in proliferating cells as shown by the lack of GFAP expression in mitotic cells in astrocytoma of all grades (Schiffer, Giordana, Germano, & Mauro, 1986) and a lack of GFAP coexpression with the proliferation marker Ki67 in 15 low grade and 10 grade IV astrocytomas (Kros, Schouten, Janssen, & van der Kwast, 1996). In contrast, low levels of proliferating GFAP‐expressing cells are detected in another study that reports coexpression of GFAP and Ki67 in 8.8% (±13.6%) of the total number of Ki67 expressing cells in low‐grade astrocytoma (Tanaka et al., 2008). In grade IV astrocytoma, Ki67 is expressed in GFAP positive cells as well, although there are significantly more Ki67 cells that are negative for GFAP (Takeuchi, Sato, Ido, & Kubota, 2006). Similarly, lower numbers of argyrophilic nucleolar organizer regions (Ag‐NORs), an indicator of proliferation rate, are present in GFAP positive cells compared to GFAP negative cells in astrocytoma of all grades (Kajiwara et al., 1992). This is confirmed in a second study that also shows a significantly lower number of Ag‐NORs in GFAP positive cells compared to GFAP negative cells, although the number of Ag‐NORs in GFAP positive cells was highly variable (Hara et al., 1991; Kajiwara et al., 1992). GFAP positive cells with high numbers of Ag‐NORs were described as well (Kajiwara et al., 1992). In contrast, a more recent study shows Ki67‐GFAP coexpression in astrocytoma and describes that 97% of Ki67 positive cells in low grade and 74% in high‐grade astrocytoma is GFAP positive. The number of Ki67‐GFAP double positive cells is significantly higher in low‐grade astrocytoma compared with high‐grade astrocytoma (L. Yang et al., 2014). According to these studies, GFAP is expressed in both proliferating and nonproliferating cells. Local differences in the distribution of these cells might again account for the variation in observations. As mentioned previously, GFAP is expressed at different levels in the core, intermediate transitional area, and periphery of the tumor (Pistollato et al., 2010). Analysis of stem cell and proliferation markers in these areas shows that the core mainly consists of CD133 positive and O^6^‐methylguanine‐DNA methyltransferase promoter methylation positive (MGMT+) stem cells that can form small neurospheres in culture. The highest level of Ki67 expression is observed in the intermediate area and these cells show the highest growth potential in vitro. Cells in the periphery show the lowest Ki67 expression and lowest growth potential in vitro. These cells do not grow as neurospheres but show a differentiated morphology. In vitro*,* GFAP expression does not significantly differ between cells cultured from the different areas of a tumor, although a trend for increased GFAP expression in peripheral cells is observed (Pistollato et al., 2010). Another study reports on coexpression of the stem cell marker CD133 with GFAP (Tamura et al., 2013) further emphasizing that GFAP expression does not solely mark proliferating or nonproliferating, differentiated or stem‐cell like cells in astrocytoma patient material.

Three studies determined GFAP expression in invading parts of astrocytoma. In areas of grade IV astrocytoma that invade cortical and white matter tissue, both GFAP positive and GFAP negative cells are present (Schiffer et al., 1986). In addition, invading astrocytoma cells into connective tissue (i.e., meningeal invasion) are marked by increased GFAP expression in the invading part compared to the noninvading part (Herpers, Budka, & McCormick, 1984; Nakopoulou et al., 1990). These studies further emphasize the large variation in GFAP expressing cells in astrocytoma that most likely causes the lack of a strong correlation of GFAP to astrocytoma malignancy based on current published literature. The identification of GFAP expressing subtypes of cells will be necessary to determine the role of GFAP in astrocytoma malignancy.

### 3.4 Differential GFAP isoform expression to distinguish astrocytoma subtypes

In the healthy human brain, the GFAP positive subtype of neurogenic stem‐cell like cells can be distinguished by the expression of a GFAP isoform that results from the process of alternative splicing, GFAPδ (Middeldorp & Hol, 2011; Roelofs et al., 2005; van den Berge et al., 2010). A few studies have determined the expression level of the GFAPδ isoform in astrocytoma of different grades as well. The studies in Table 7 report that GFAPδ expression is rarely observed in the healthy brain (Choi et al., 2009; Heo et al., 2012), but is detected in reactive gliosis (Andreiuolo et al., 2009), and is increased in astrocytoma of all grades when analyzed by immunohistochemistry (Choi et al., 2009; Heo et al., 2012). In contrast, RNA quantification of GFAPδ shows increased levels in only three out of eight and decreased levels in five out of eight grade IV astrocytoma compared to control tissue (Blechingberg et al., 2007). Interestingly, studies that find differences in GFAP expression between astrocytoma grades report on a decrease of general GFAP levels with increasing astrocytoma grade, but higher levels of GFAPδ are found in grade IV astrocytoma compared to grade I (Andreiuolo et al., 2009), grade II (Brehar et al., 2014), and grade I, II, and III (Choi et al., 2009). In one of these studies, general GFAP levels are quantified and show a decrease in grade IV compared to grade III, II, I, and control tissue (Choi et al., 2009). In grade I, II, and III spinal cord astrocytoma, GFAPδ expression also increases with increasing grade (Heo et al., 2012). Interestingly, GFAPδ levels are significantly associated with a rounder cell morphology and fewer cellular processes (Choi et al., 2009; Heo et al., 2012), and with highly invasive grade IV astrocytoma (Brehar et al., 2014). In addition, two grade II astrocytoma that are categorized as highly invasive show strong GFAPδ expression (Brehar et al., 2014). These studies indeed indicate that GFAPδ can be used to identify astrocytoma subpopulations of cells as well and suggest that GFAP expressing cells with different functions (e.g., proliferating, quiescent, invasive, and static) consist of a different combination of GFAP protein isoforms. The detection of a second GFAP alternative splice variant, GFAPκ, in RNA isolated from grade IV astrocytoma further supports this hypothesis (Blechingberg et al., 2007). We recently showed that quantification of the relative level of GFAPδ to GFAPα, the GFAPδ/GFAPα ratio, using RNA sequencing data obtained from the cancer genome atlas (TCGA) indeed indicates that low‐ and high‐grade astrocytoma express different combinations of GFAP variants. In astrocytoma grade IV, the GFAPδ/GFAPα ratio was significantly higher compared to grade II and III (WHO 2007; Stassen et al., 2017).

### 3.5 GFAP as a blood biomarker

The most consistent results on the relationship of GFAP to astrocytoma malignancy have been generated by the analysis of blood or cerebrospinal fluid (CSF) of astrocytoma patients. An early study already shows that GFAP levels in CSF can be used to distinguish astrocytoma from other types of tumors and healthy controls (Szymas, 1985), but there was no follow up study. As shown in Table 8, subsequent studies link GFAP detection in blood to grade IV astrocytoma specifically. Eight out of 12 studies report on a significant association of GFAP levels detected in serum (Baumgarten et al., 2018; Gállego Pérez‐Larraya et al., 2014; Ilhan‐Mutlu et al., 2013; Jung et al., 2007; Kiviniemi et al., 2015; Tichy et al., 2016), in microparticles (Sartori et al., 2013), and in mono‐nucleated cells (Muller et al., 2014) isolated from blood of grade IV astrocytoma patients in comparison to lower grade astrocytoma, nonglial tumors, other neurological diseases, and healthy controls. One of these studies reports detectable GFAP levels in blood of grade III astrocytoma patients as well, but levels in grade IV astrocytoma patients were significantly higher (Kiviniemi et al., 2015). Similarly, GFAP positive exosome numbers are increased in grade III and IV astrocytoma compared to healthy controls (Galbo et al., 2017). Two of the 12 studies report on higher GFAP levels in plasma of grade IV patients, but no statistics were performed (Vietheer et al., 2017) or no statistical significance was reached (Lange et al., 2014). High GFAP levels in blood of grade IV astrocytoma patients is in contrast with the most often described lower GFAP levels in higher grade astrocytoma tissue (Tables 3, 4, 5). However, although one study also finds higher GFAP expression levels in tumor cells of patients with high GFAP serum levels (Tichy et al., 2016), another study reports on the absence of a correlation (Kiviniemi et al., 2015) suggesting that GFAP serum levels not directly reflect GFAP expression in the tumor. Indeed, multiple other tumor‐related factors are associated with increased GFAP serum levels. A significant correlation to tumor volume and/or necrosis (Gállego Pérez‐Larraya et al., 2014; Jung et al., 2007; Kiviniemi et al., 2015) and the number of GFAP positive necrotic cells (Jung et al., 2007) has been described, although in other studies a correlation to tumor volume was assessed but absent (Baumgarten et al., 2018; Ilhan‐Mutlu et al., 2013; Vietheer et al., 2017). In addition, increased GFAP serum levels are observed up to 7 days after surgical removal of grade IV and III astrocytoma (Baumgarten et al., 2018; Kiviniemi et al., 2015). These studies suggest that GFAP serum levels are related to brain damage and cell death induced by, in, or near the tumor. This is supported by studies that have linked high GFAP serum levels in patients with traumatic brain injury (Bazarian et al., 2018; Thelin et al., 2017). Increased levels of GFAP positive microparticles can be observed from 7 days up to 7 months after surgery (Sartori et al., 2013), although another study that measured GFAP serum levels 6 weeks after surgery, does not show an increase in GFAP and for some patients the GFAP serum levels were even lower compared to levels before surgery (Vietheer et al., 2017). GFAP levels in blood might be induced by regrowth of the tumor, as levels of GFAP microparticles at 7 months compared to 7 days are increased in blood of patients with subtotal compared to gross‐total resections of the tumor (Sartori et al., 2013). Moreover, increased levels after 7 months of surgery compared to 4 months are seen in patients with radiological disease progression (Sartori et al., 2013). Furthermore, the significant negative correlation of preoperative GFAP serum levels with the time until tumor recurrence (progression‐free survival [PFS]) for grade III and IV astrocytoma patients (Kiviniemi et al., 2015) indicates that factors in the biology of the tumor contribute to the GFAP serum levels, rather than surgical damage only. Although, most studies did not find a significant correlation to either progression‐free survival or survival of patients (Gállego Pérez‐Larraya et al., 2014; Ilhan‐Mutlu et al., 2013; Jung et al., 2007; Muller et al., 2014; Sartori et al., 2013; Vietheer et al., 2017). In addition, high GFAP levels in blood of patients prior to treatment are associated with epidermal growth factor receptor amplified (EGFRvIII) tumors within grade IV astrocytoma (Muller et al., 2014), with IDHwt tumors within grade III and IV astrocytoma (Kiviniemi et al., 2015) and with higher levels of Ki67 (cell division marker) expression in tumor cells (Kiviniemi et al., 2015). Interestingly, one study has isolated GFAP expressing cells from blood of grade IV astrocytoma patients and showed that they contain astrocytoma specific mutations, indicating that GFAP positive glioma cells can leave the tumor and enter the bloodstream (Muller et al., 2014). Possibly, there is a specific subpopulation of GFAP expressing cells in grade IV glioma that is well‐equipped to enter the bloodstream and GFAP isoform expression might be used to further characterize this subpopulation of cells that are associated with a higher malignant astrocytoma. Nonetheless, regardless of the cause of GFAP protein in serum of glioma patients, that is, damage, specific factors in the biology of the tumor, or the entrance of glioma cells into the circulation, measurements of GFAP levels in serum might be useful in diagnosis of grade IV astrocytoma and prognosis in relation to the progression of the disease. Distinguishing between GFAP isoforms to improve the diagnostic capacity of serum GFAP is an interesting approach that should be taken in the future.

## 4 CONCLUSION

**Figure 1:**
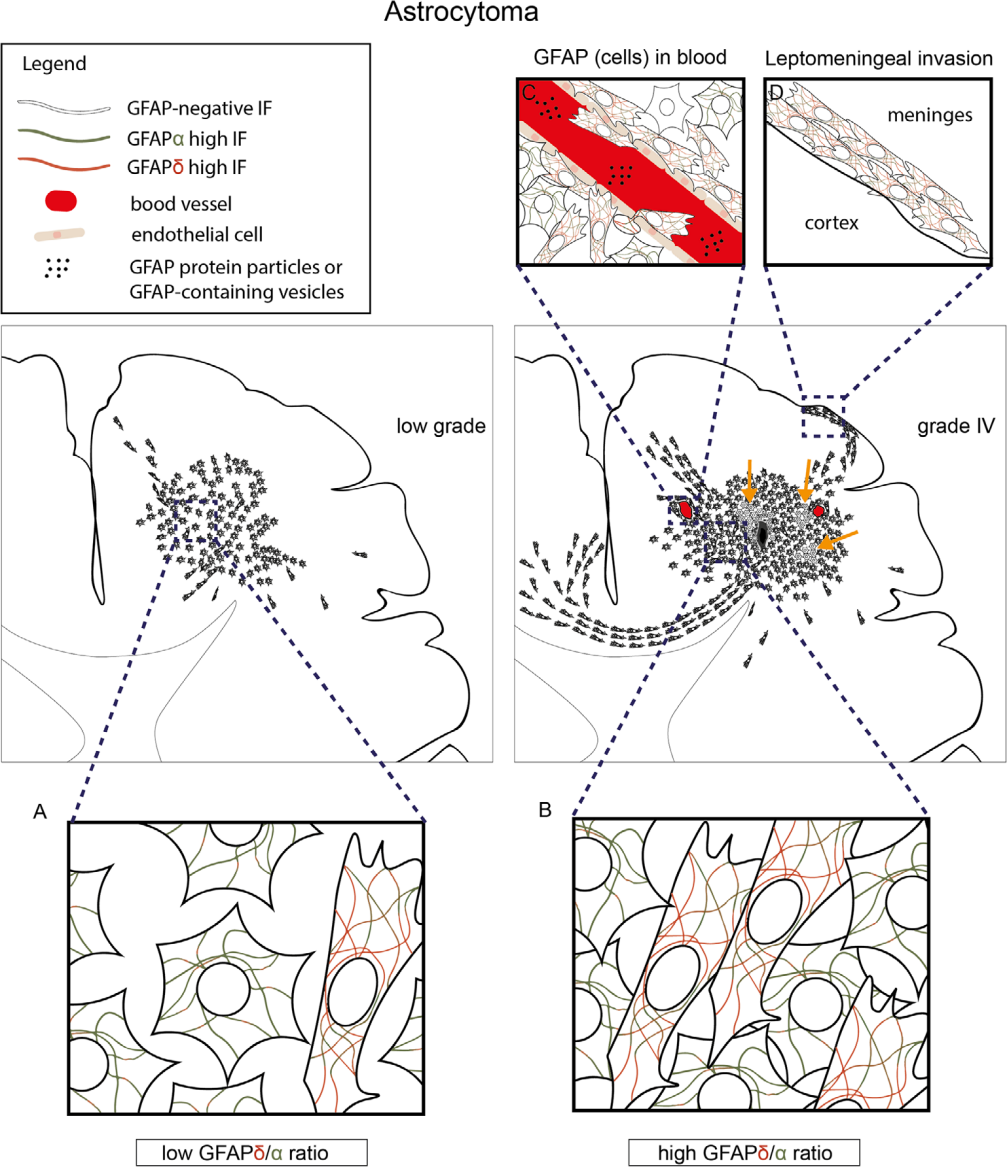
The GFAPδ/α ratio distinguishes astrocytoma subpopulations. Overview of low‐ (left panel) and high‐grade (right panel) astrocytoma and differences in the heterogeneous GFAP positive cell population. High‐grade astrocytoma (right panel) is characterized by increased mitosis and cell density, necrosis (black area) and vascularization (red vessels). Invasive astrocytoma cells use white matter tracts, blood vessels and meninges as a surface to migrate on (Claes, Idema, & Wesseling, 2007). GFAP levels in blood are specifically associated with grade IV astrocytoma. In both high‐ and low‐grade astrocytoma, the GFAP positive cell population is highly heterogeneous and contains cells with various functions (e.g., proliferating, quiescent, invasive, and static). GFAP negative areas are more often found in the center of high‐grade tumors (orange arrows). The GFAPδ isoform distinguishes astrocytoma subpopulations of cells (a, b), and as the GFAPδ/α ratio is increased in grade IV astrocytoma, this subpopulation is most likely larger in these tumors (b). GFAP protein and GFAP positive cells in blood of patients are associated with high‐grade astrocytoma and might contain different levels of GFAP isoforms (c). Similarly, invading cells that, for example, invade the meninges (connective tissue) might consist of a specialized GFAP network that equips them for this behavior (d)

GFAP positive cells are present in tumors of all malignancy grades with a tendency for decreased GFAP levels with increasing astrocytoma grade. However, in current literature, a significant correlation is not consistently reproduced mainly caused by intra‐ and inter‐tumor heterogeneity of GFAP positive cell localization, morphology, function, and expression of GFAP variants. Different types of evidence support the presence of a specialized GFAP intermediate filament network composed of different GFAP variants (splice isoforms, posttranslational modifications, degradation products) in astrocytoma cell subpopulations. As these variants, as shown for GFAPδ, differentially correlate to the malignancy of the tumor, the current use of commercial GFAP antibodies that recognize all isoforms most likely masks a consistent correlation of GFAP to astrocytoma malignancy grade. Discrimination between GFAP variants, as we show here for GFAPδ and GFAPα, helps to identify different types of GFAP positive cells that could improve the assessment of astrocytoma differentiation and malignancy. We hypothesize, as summarized in Figure 1, that GFAP is expressed in heterogenous astrocytoma cells with a low malignant, more differentiated and noninvasive phenotype, as well as a high malignant, stem‐cell like more invasive phenotype. Higher levels of GFAPδ are expressed in neurogenic stem‐cell like cells of the healthy brain (Middeldorp & Hol, 2011; Roelofs et al., 2005; van den Berge et al., 2010) and in higher malignant astrocytoma (Andreiuolo et al., 2009; Brehar et al., 2014; Choi et al., 2009; Heo et al., 2012), and the GFAPδ/α ratio is increased in grade IV astrocytoma (Stassen et al., 2017). Therefore, cells with a high GFAPδ/α ratio might be the high malignant, stem‐cell like more invasive cells of the GFAP cell population. These cells are present at lower numbers in low‐grade astrocytoma and could potentially induce progression into higher malignancy grades. Differences in malignant behavior of cells with a high and low GFAPδ/α ratio support this hypothesis (Moeton et al., 2014; Stassen et al., 2017) and future studies should focus on unravelling the isoform‐specific function in astrocytoma malignancy. In conclusion, information is lost when the expression of different GFAP isoforms is neglected and can be deceiving when GFAP is used to determine the differentiation state of a cell in experimental and clinical settings. Therefore, future studies need to focus on further identifying the GFAP positive cell population and make use of the possibility to discriminate between GFAP variants that could be fruitful to diagnosis and to the understanding of the molecular basis of glioma.

## CONFLICTS OF INTEREST

The authors declare no conflicts of interest.
